# Persistent descending mesocolon as a vital risk factor for anastomotic failure and prolonged operative time for sigmoid colon and rectal cancers

**DOI:** 10.1186/s12957-023-03091-w

**Published:** 2023-07-07

**Authors:** Shiwen Mei, Mingguang Zhang, Feng Ye, Wenlong Qiu, Jichuan Quan, Meng Zhuang, Xishan Wang, Jianqiang Tang

**Affiliations:** 1 Department of Colorectal Surgery, National Cancer Center/National Clinical Research Center for Cancer/Cancer Hospital, Chinese Academy of Medical Sciences and Peking Union Medical College. No, 17 Panjiayuan Nanli, Chaoyang District, Beijing, 100021 China; 2 Department of Radiology, National Cancer Center/National Clinical Research Center for Cancer/Cancer Hospital, Chinese Academy of Medical Sciences and Peking Union Medical College, No.17 Panjiayuan Nanli, Chaoyang District, Beijing, 100021 China

**Keywords:** Persistent descending mesocolon, Anterior resection, Laparoscopic surgery, Multiplanar reconstruction, Maximum intensity projection, Anastomotic failure

## Abstract

**Background:**

The diagnostic criteria and effect of persistent descending mesocolon (PDM) on sigmoid and rectal cancers (SRCs) remain controversial. This study aims to clarify PDM patients' radiological features and short-term surgical results.

**Method:**

From January 2020 to December 2021, radiological imaging data from 845 consecutive patients were retrospectively analyzed using multiplanar reconstruction (MRP) and maximum intensity projection (MIP). PDM is defined as the condition wherein the right margin of the descending colon is located medially to the left renal hilum. Propensity score matching (PSM) was used to minimize database bias. The anatomical features and surgical results of PDM patients were compared with those of non-PDM patients.

**Results:**

Thirty-two patients with PDM and 813 patients with non-PDM were enrolled into the study who underwent laparoscopic resection. After 1:4 matching, patients were stratified into PDM (*n* = 27) and non-PDM (*n* = 105) groups. The lengths from the inferior mesenteric artery (IMA) to the inferior mesenteric vein (1.6 cm vs. 2.5 cm, *p* = 0.001), IMA to marginal artery arch (2.7 cm vs. 8.4 cm, *p* = 0.001), and IMA to the colon (3.3 cm vs. 10.2 cm, *p* = 0.001) were significantly shorter in the PDM group than those in the non-PDM group. The conversion to open surgery (11.1% vs. 0.9%, *p* = 0.008), operative time (210 min vs. 163 min, *p* = 0.001), intraoperative blood loss (50 ml vs. 30 ml, *p* = 0.002), marginal arch injury (14.8% vs. 0.9%, *p* = 0.006), splenic flexure free (22.2% vs. 3.8%, *p* = 0.005), Hartmann procedure (18.5% vs. 0.0%, *p* < 0.001) and anastomosis failure (18.5% vs. 0.9%, *p* = 0.001) were significantly higher in the PDM group. Moreover, PDM was an independent risk factor for prolonged operative time (OR = 3.205, *p* = 0.004) and anastomotic failure (OR = 7.601, *p* = 0.003).

**Conclusion:**

PDM was an independent risk factor for prolonged operative time and anastomotic failure in SRCs surgery. Preoperative radiological evaluation using MRP and MIP can help surgeons better handle this rare congenital variant.

## Introduction

Persistent descending mesocolon (PDM) is a congenital disease first reported by Morgenstern in 1960 [1]. The incidence of PDM varies from 1.3% to 4.0% [2–4]. For patients with PDM, the descending colon is often medially located toward the failed fusion with the dorsal abdominal wall. The sigmoid colon is shifted to the right side of the abdomen with adhesion to the intestine mesentery. Some clinical complications, such as colonic volvulus, primary intestinal obstruction, and internal hernia, were reported in patients with PDM [5]; however, most patients are asymptomatic. PDM diagnosis mainly relies on imaging examinations, such as barium enema and abdominal CT scans [6–8]; moreover, it can also be identified incidentally during surgery.

The current PDM and colorectal surgery research is mostly case-by-case [9–11]. Although several retrospective studies are available, they have not been able to present the effect of PDM on anterior resection or low anterior resection (AR/LAR) due to left hemicolectomy enrollment. In addition, whether the variant characteristics of PDM affect short-term outcomes and colorectal anastomosis still lacks data.

Hence, this study focused on patients with PDM undergoing radical surgery for sigmoid and rectal cancers (SRCs). The anatomic relationships of the inferior mesenteric artery (IMA), the inferior mesenteric vein (IMV), the left colic artery (LCA), and the colon were evaluated using multiplanar reconstruction (MPR) and maximum intensity projection (MIP) techniques of CT post-processing [12]. In addition, the effects of PDM on short-term surgical outcomes and anastomotic outcomes of SRCs were further evaluated.

## Materials and methods

### Patients

**Fig. 1:**
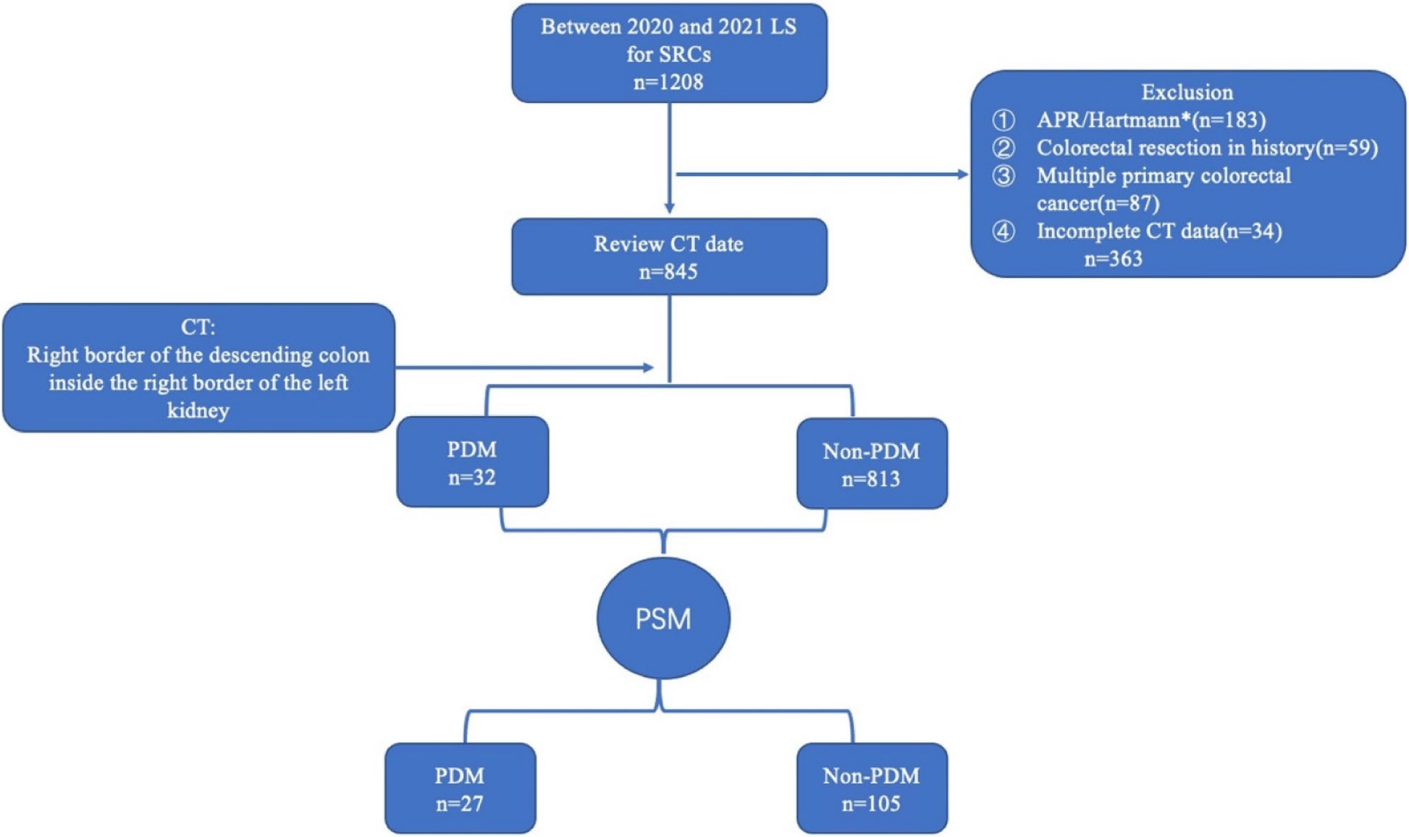
Flow-chart. LS, Laparoscopic surgery; SRCs, sigmoid and rectal cancers; APR, abdominoperineal resection; PDM, persistent descending mesocolon;PSM, Propensity Score Matching

A total of 1208 patients with SRCs who underwent radical surgery for SRCs via laparoscopy at the Department of Colorectal Surgery, Cancer Hospital Chinese Academy of Medical Sciences, were retrospectively collected between January 2020 and December 2021. The inclusion criteria were as follows: (1) having SRC tumors; (2) having adenocarcinoma, as confirmed by pathological biopsy; (3) having undergone radical resection using laparoscopy; and (4) having undergone preoperative enhanced abdominal and pelvic CT. The exclusion criteria were: (1) having undergone abdominal perineal resection (APR); (2) having taken an initial decision to perform the Hartmann's procedure; (3) having colorectal surgery history; (4) having multiple primary tumors in the colon and rectum; and (5) having incomplete radiological imaging data. The flow chart is presented in Fig. 1.

## Data collection

Data relating to patient demographics, clinicopathological features, radiological findings, and perioperative characteristics were reviewed retrospectively, including age; gender; body mass index (BMI); ASA Physical Status (ASA); tumor location, TNM staging and carcinoembryonic antigen (CEA) level; receiving of neoadjuvant chemoradiotherapy (nCRT), LCA retention and indocyanine green (ICG) angiography. Furthermore, radiological findings included the IMA bifurcating patterns; the lengths between the IMA and IMV, IMA and LCA, IMA and marginal arch, and IMA and the colon; and the length of IMA and IMA shift (right or left). Perioperative characteristics included operative time, blood loss, surgical procedure, intraoperative accident, number of lymph nodes harvested, conversion to laparotomy, anastomotic failure, postoperative complications, mortality, time-to-first anal exhaust, time-to-first oral intake, and the length of hospital stay.

### Definition and radiological findings of PDM

**Fig. 2:**
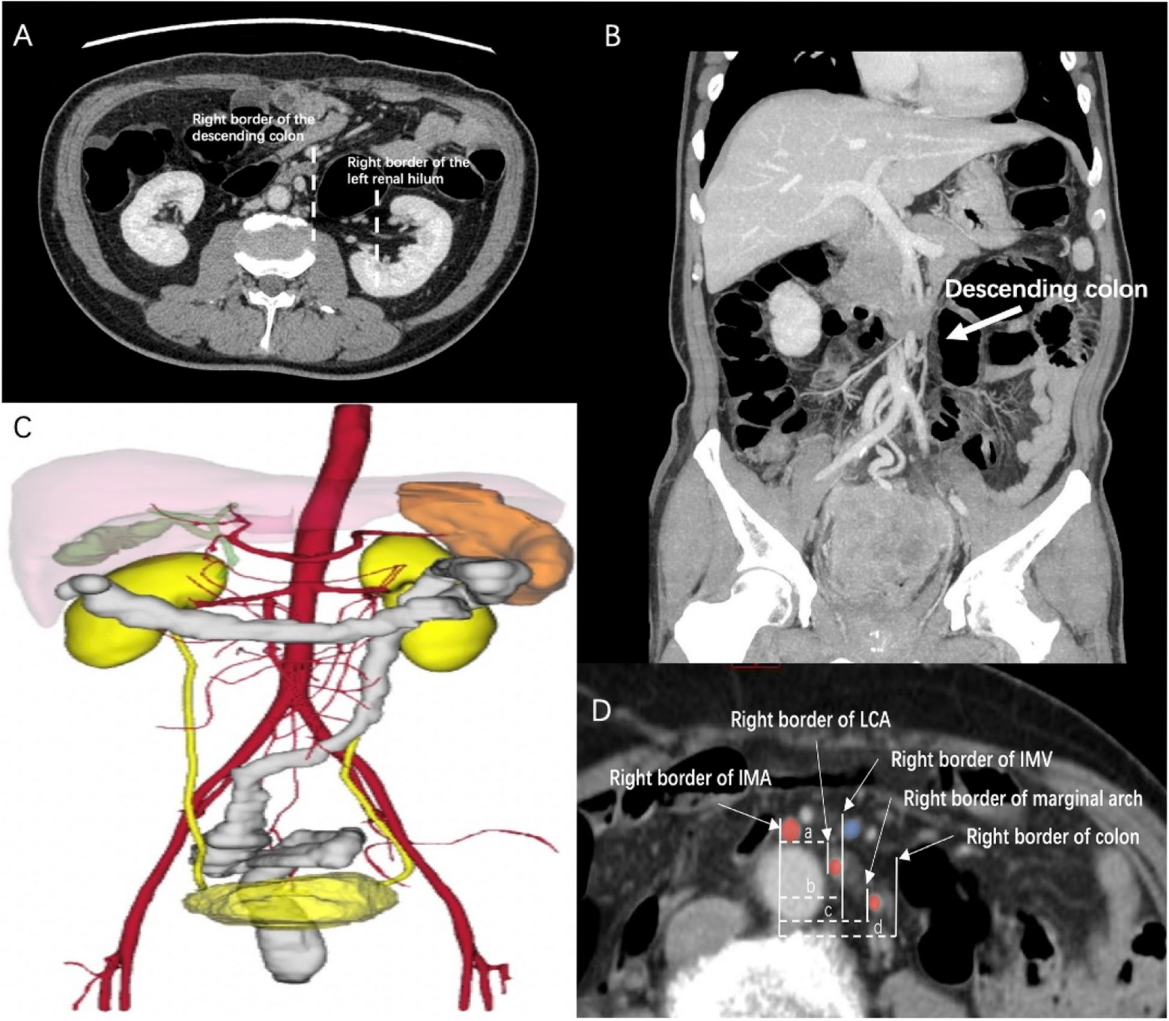
The characteristics of PDM in the CT scan. **A** CT Axis, PDM was defined as “the right margin of the descending colon located in the left renal hilum.” **B** coronal CT scan: the descending colon near the midline of the abdomen. **C** 3D reconstruction: the sigmoid colon shifted to the right side of the abdomen. **D **Measurement of various lengths at the IMA root level: a. length between the IMA and LCA. b. length between the IMA and IMV c. length between the IMA and marginal arch. d. length between the IMA and the colon

Each patient underwent a preoperative enhanced abdominal and pelvic CT scan. According to the CT diagnosis, PDM was confirmed as the right margin of the descending colon being located medially to the left renal hilum [4]. MPR is suitable for structural imaging on any plane, and MIP can project the voxel with the maximum CT value in a certain thickness (CT layer thickness) onto the background plane to display all or part of the vessels and (or) organs with high enhancement density. MPR helped obtain coronary position images, and MIP (10 mm thickness) was used to confirm the IMA root location and bifurcating patterns (Fig. 2A–C). The length of the IMA (the distance from the IMA root to its first branch) and the distances between the IMA and IMV, the IMA and LCA (the distance from the IMA root to its same level LCA branch), the IMA and the marginal arch, and the IMA and the descending colon were measured on axial images. Furthermore, the right edge of both vessels was measured as the starting and ending point (Fig. 2D). The branching pattern of the IMA was classified into four types based on a previously published classification as follows: type I: the LCA arises from the IMA independently of the sigmoid artery, type II: the LCA and first SA have a common trunk, type III: radial-branching of the LCA, SA, and SRA from the IMA, and type IV: no LCA [13].

### Surgical procedures

After general anesthesia induction, the patient was placed in modified lithotomy, 30° Trendelenburg position, and five ports were placed as per routine practice for laparoscopic proctectomy. A pneumoperitoneum was created using 14 mmHg pressure. We determined the location of the descending and sigmoid colons. The descending colon was located medially to the left renal hilum, the site of adhesion with the pelvic wall, and the ileocecal area and its congenital adhesion. First, the adhesion between the sigmoid colon and the ileocecal mesentery was released to expose the IMA area. Subsequently, the right yellow-white junction line was cut into the retrorectal space and was fully exposed to the opposite side. After joining the left and right sides, we bound the rectum and mesentery using a sling, lifted it from a caudal to a cranial direction to expand the retrorectal space, and finally reached the IMA root [14]. The preservation of LCA and the decision to free the splenic flexure were determined based on the intraoperative situation and preferences of the surgeon. Splenic flexure free was required for patients with short length of sigmoid colon, high anastomotic tension or intraoperative marginal vascular arch injury, and LCA preservation was usually proposed in the elderly, diabetes patients and other insufficient blood supply diseases.

**Fig. 3:**
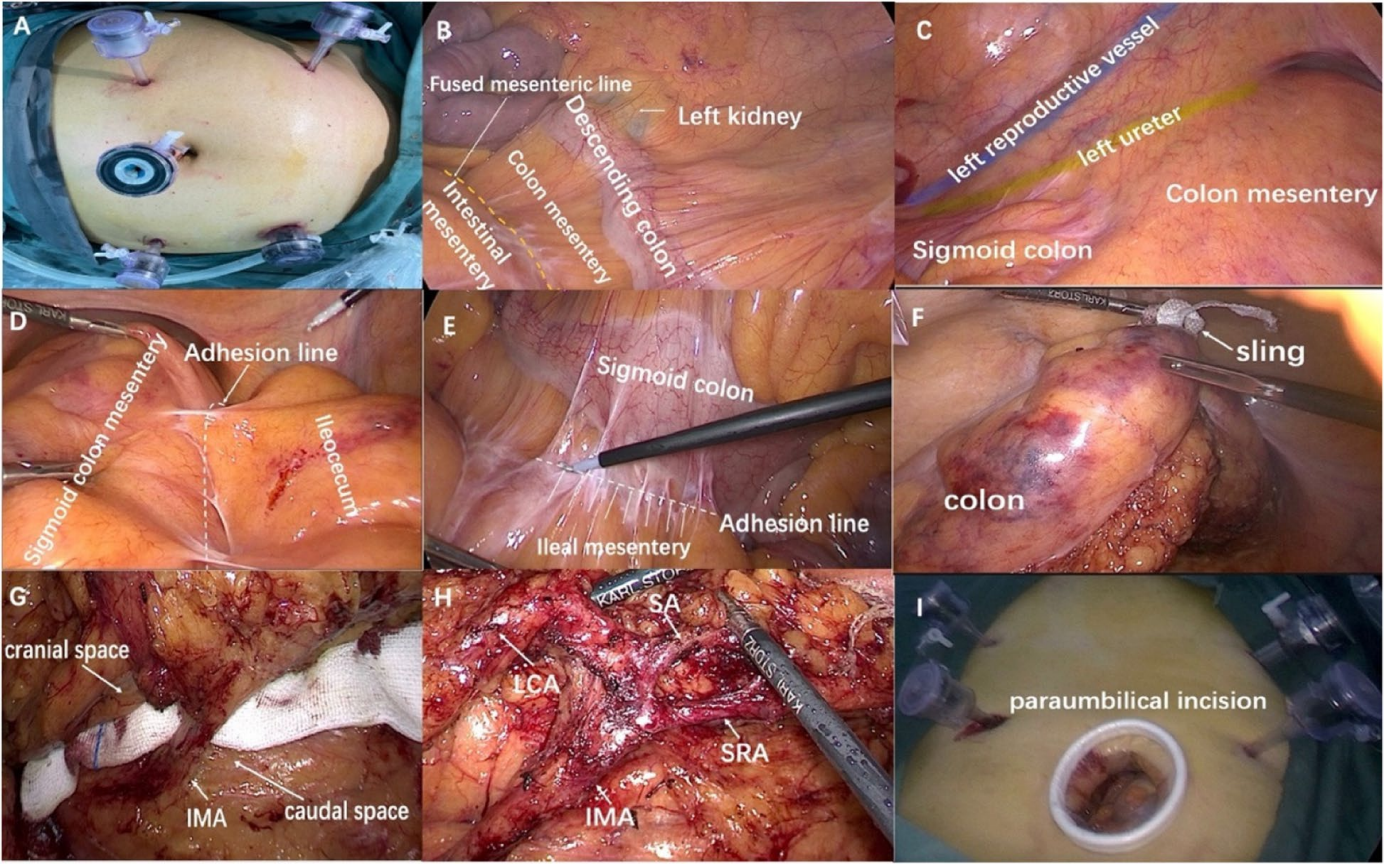
**A** Trocars position. **B** The descending colon was on the medial side of the left kidney. **C** The congenital adhesion of the sigmoid colon was lost to the left abdominal wall, and the left ureter and reproductive vessels were exposed outside the mesentery. **D** The sigmoid colon mesentery adhered to the ileocecum. **E** The sigmoid colon adhered to the ileal mesentery to block IMA exposure. **F** Take a sling to bind the rectum and mesentery and lift it from the caudal to the cranial to expand the retro rectal space. **G** IMA exposure through a cephalic intermediate approach. **H** The IMA branches were dissected and classified. **I** The specimens were taken out through a paraumbilical incision

When the mesentery was shaped, the marginal arch was cautiously preserved to avoid colonic ischemia, and the specimens were extracted through a paraumbilical or supraumbilical incision on the cephalad side. The colorectal anastomosis was performed intracorporeally (Fig. 3A-I). The Hartmann procedure was “mandatorily performed due to marginal arch injury or failure of free congenital adhesion.” The operative time, intraoperative blood loss, conversion to open or Hartmann procedure, marginal arch injury and splenic flexure free were recorded.

### Postoperative outcomes and follow-up

Postoperative conditions, including time-to-first anal exhaust, time-to-first oral intake, complications, postoperative recovery, number of lymph nodes harvested, TNM staging, and hospitalization days, were recorded. The Clavien-Dindo classification was used to evaluate the severity, and patients were followed up regularly [15]. Tumor markers were measured at each visit. In addition, CT scans of the abdomen and chest were obtained generally every six months.

### Statistical analysis

To reduce the imbalance between the groups, PSM was performed 1:4 to match patients in PDM group with those in non-PDM group (caliper = 0.2). We selected baseline date observed in the overall cohort or variables that may interfere with the results as co-variables for matching, and the covariates included age, gender, BMI, ASA score, tumor location, TNM staging, carcinoembryonic antigen (CEA) level, receiving of neoadjuvant chemoradiotherapy (nCRT), LCA retention and ICG angiography. The Chi-square test was used for categorical variables, and Fisher’s exact test was performed. For continuous variables, we first checked for normal distribution, and then the T-test was used to compare the differences between both groups. Finally, the U test was used to compare the differences between both groups for variables that did not conform to normal distribution. A *p*-value of < 0.05 was considered significant, and logistic regression analysis was performed. All the data were statistically analyzed using Statistical Product Social Sciences (SPSS 26.0) and RStudio (2022.07.2) for mac.

## Results

### Patient characteristics

According to PDM diagnostic criteria, the patients were divided into the PDM (32/845, 3.8%) and the non-PDM group (813/845, 96.2%). 228(28.2%) patients were aged ≥ 60 years, and 411 (48.6%) were male. Patients with BMI ≥ 25 kg/m^2^ were 327(38.7%) in the entire cohort. Tumors were located in the rectum and sigmoid in 55.3% and 44.7% of cases, respectively. 127 patients (15.0%) received preoperative chemoradiotherapy. The patients’ other baseline characteristics are described in Table 1.

**Table 1:** Patient characteristics

Variables	Total cohort			Matched cohort		
	PDM (*n* = 32)	Non-PDM (*n* = 813)	*P*	PDM (*n* = 27)	Non-PDM (*n* = 105)	*p*
Age			0.839			1.000
≥ 60	15 (46.9%)	396 (48.7%)		10 (37.0%)	40(38.1%)	
< 60	17 (53.1%)	417 (51.3%)		17(63.0%)	65(61.9%)	
Gender			0.117			0.748
Male	24 (76.0%)	498 (61.3%)		19(70.4%)	68(64.8%)	
Female	8 (24.0%)	315 (38.7%)		8(29.6%)	37(35.2%)	
BMI (kg/m^2^)			0.820			0.811
≥ 25	32 (40.6%)	314 (38.6%)		10 (37.0%)	44(41.9)	
< 25	19 (59.4%)	499 (61.4%)		17(63.0%)	61(58.1)	
ASA score			0.822			0.988
I/II	28 (84.0%)	700 (86.1%)		23(85.2)	92(87.6)	
III	4 (16.0%)	113 (13.9%)		4(14.8)	13(12.4)	
Tumor location			0.011			0.936
Rb	12 (32.0)	137 (16.9)		7(25.9)	30(28.6)	
Ra	9 (32.0)	309(38.0)		9(33.3)	36(34.3)	
Rs	11(36.0)	367 (45.1)		11(40.7)	39(37.1)	
pTNM			0.804			0.936
I	9 (28.0%)	194 (23.9%)		8(29.6)	34(32.4)	
II	10 (40.0%)	235 (28.9%)		7(25.9)	29(27.6)	
III	12 (28.0%)	347 (42.7%)		11(40.7)	40(38.1)	
IV	1 (4.0%)	37 (4.5%)		1(3.7)	2(1.9)	
CEA			0.688			0.390
≥ 5 ng/L	9 (28.1%)	256 (31.5%)		7(25.9)	18(18.8)	
< 5 ng/L	23 (71.9%)	557 (68.5%)		20(74.1)	78(81.2)	
nCRT	5 (8.0%)	122 (15.0%)	0.923	5(18.5)	18(17.1)	1.000
LCA retention	14(43.8)	104(12.8)	0.001	9(33.3)	32(30.5)	0.958
ICG						

*PDM*: Persistent descending mesocolon, *BMI* Body mass index, *ASA* American Society of Anesthesiologists, *CEA* Carcinoembryonic antigen, *nCRT* Neoadjuvant chemoradiotherapy, *LCA* Left colic artery, *Ra* Rectum(above the peritoneal reflection), *Rb* Rectum(below the peritoneal reflection), *Rs* Rectosigmoid

Patients’ characteristics before and after matching between the groups are presented in Table 1. Before PSM, compared with the PDM group, the non-PDM group had fewer female patients (*p* = 0.117). Furthermore, the proportion of tumor location (Rb,Ra,Rs) in PDM group and non-PDM group were different (*p* = 0.011). After PSM, the PDM and non-PDM groups were well balanced in terms of the above-mentioned variables (*p* > 0.05).

## Radiological findings

**Fig. 4:**
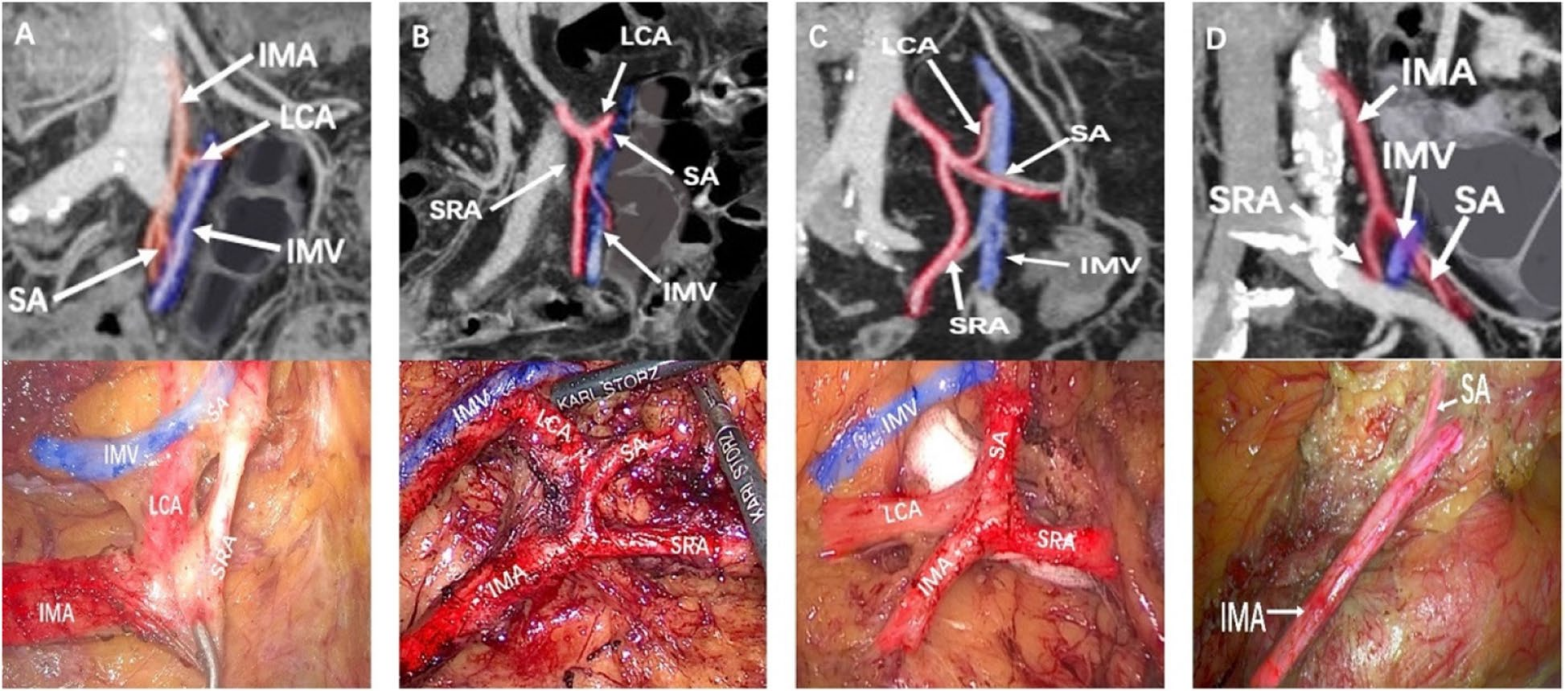
Intraoperative bifurcating IMA Patterns in the coronal section of MRP-MIP. **A** type I: LCA arises independently from IMA; **B** type II: LCA and SA were given off at the same point; **C** type III: LCA and SA were branched from a common trunk from IMA; **D** type IV: LCA was lacking

Radiological findings are presented in the total and matched cohorts are summarized in Table 2. Before matching, the frequency of types I, II, III, and IV in the PDM and non-PDM groups was 37.5%/31.3%/28.1%/3.1% and 47.1%/30.1%/17.6%/5.1%, respectively (Fig. 4A-D). The PDM group had higher rates of IMA originating from the right side of the aorta than did the non-PDM group (40.0% vs. 1.0%, *p* < 0.001).The lengths between IMA and IMV (1.6 vs. 2.5 cm, *p* < 0.001), IMA and LCA (1.5 vs. 1.6 cm, *p* = 0.063), IMA and the marginal artery arch (2.7 vs. 8.4 cm, *p* < 0.001), and IMA and the colon (3.5 vs. 9.8 cm, *p* < 0.001) were significantly shorter in the PDM group than those in the non-PDM group.

**Table 2:** Radiological findings

Variable	Total cohort			Matched cohort		
	PDM (*n* = 32)	Non-PDM (*n* = 813)	*P*	PDM (*n* = 27)	Non-PDM (*n* = 105)	*P*
Branching Patterns of IMA			0.430			0.681
I	12 (37.5%)	384 (47.2%)	0.279	11(40.7%)	42(40.0%)	0.994
II	10 (31.3%)	245 (30.1%)	0.893	8(29.7%)	36(34.3%)	0.647
III	9 (28.1%)	143 (17.6%)	0.128	7(25.9%)	19(18.1%)	0.417
IV	1 (3.1%)	41 (5.1%)	0.624	1(3.7%)	7(6.6%)	0.685
Length between IMA and IMV (cm)	1.6 ± 0.6	2.5 ± 0.2	< 0.001	1.6 ± 0.4	2.5 ± 0.3	0.001
Length between IMA and LCA (cm)	1.5 ± 0.7	1.6 ± 0.5	0.063	1.4 ± 0.5	1.6 ± 0.3	0.083
Length between IMA and Marginal Arch (cm)	2.7 ± 0.9	8.4 ± 0.8	< 0.001	2.7 ± 0.9	8.4 ± 0.6	0.001
Length between IMA and Colon (cm)	3.5 ± 1.2	9.8 ± 2.1	< 0.001	3.3 ± 1.7	10.2 ± 1.9	0.001
Length of IMA(cm)	3.7 ± 0.8	3.7 ± 0.9	0.516	3.7 ± 1.0	3.6 ± 0.6	0.763
Shift of IMA			< 0.001			0.001
Left	22(60.0%)	805 (99.0%)		18(66.7%)	105(100.0%)	
Right	10 (40.0%)	8 (1.0%)		9(33.3%)	0(0.0%)	

*LCA* Left colic artery, *IMA* Inferior mesenteric artery, *IMV* Inferior mesenteric vein

After PSM, the frequency of types I, II, III, and IV in the PDM and non-PDM groups was 40.7%/29.7%/25.9%/3.7% and 40.0%/34.3%/18.1%/6.6%, respectively (Fig. 4A-D). The PDM group had higher rates of IMA originating from the right side of the aorta than did the non-PDM group (33.3% vs. 0.0%, *p* = 0.001). The lengths between IMA and IMV (1.6 vs. 2.5 cm, *p* = 0.001), IMA and LCA (1.4 vs. 1.6 cm, *p* = 0.083), IMA and the marginal artery arch (2.7 vs. 8.4 cm, *p* = 0.001), and IMA and the colon (3.3 vs. 10.2 cm, *p* = 0.001) were significantly shorter in the PDM group than those in the non-PDM group.

### Perioperative characteristics

The perioperative characteristics, including intraoperative data and surgical results, in total and matched cohorts are presented in Table 3. Before PSM, four patients in the PDM group and ten in the non-PDM group needed conversion to open surgery or extracorporeal IMA dissection (16.0% vs. 1.2%, *p* < 0.001). The median operative time for the PDM group was longer than that for the non-PDM group (207.5 vs. 156 min, *p* = 0.001). Furthermore, the median intraoperative blood loss in the PDM group was significantly more than that in the non-PDM group (50 vs. 30 mL, *p* = 0.006). The marginal arch injury (20.0% vs. 1.1%, *p* < 0.001), the ratio of free splenic flexure (28.0% vs. 3.9%, *p* < 0.001), and the rate of mandatory Hartmann procedure (15.6% vs. 0.1%, *p* < 0.001) were significantly higher in the PDM than those in the non-PDM group. The proportion of anastomotic failure in the PDM group was significantly higher than that in the non-PDM group (25.0% vs. 3.9%, *p* < 0.001). Moreover, anastomotic leakage (AL) incidence in the PDM group displayed an increasing tendency (9.4% vs. 3.8%, *p* = 0.113). Wound infection in PDM group were more than that in non-PDM group (6.3% vs. 0.6%, *p* = 0.026).

**Table 3:** Perioperative characteristics

Variable	Total cohort			Matched cohort		
	PDM (*n* = 32,%)	Non-PDM (*n* = 813,%)	*P*	PDM (*n* = 27)	Non-PDM (*n* = 105)	*P*
Operative time	207.5(140,240)	156(121,189)	0.001	210(142,240)	163(125,195)	0.001
Blood loss	50(20,100)	30(20, 50)	0.006	50(20,100)	30(20,50)	0.002
Surgery procedure			< 0.001			< 0.001
Colorectal anastomosis	27 (84.4%)	812 (99.9%)		22(81.5%)	105(100.0%)	
Hartmann^*^	5 (15.6%)	1 (0.1%)		5(18.5%)	0 (0.0%)	
Intraoperative accident						
Marginal Arch injury	5 (20.0%)	9 (1.1%)	< 0.001	4(14.8%)	1(0.9%)	0.006
Splenic flexure free	7 (28.0%)	27 (3.3%)	< 0.001	6(22.2%)	4(3.8%)	0.005
Lymph nodes harvested	17 (14, 24)	20 (15, 26)	0.192	18(15,24)	21(14,25)	0.006
Conversion	4 (16.0%)	10 (1.2%)	< 0.001	3(11.1%)	1(0.9%)	0.008
Anastomosis failure^a^	7 (28.0%)	32 (3.9%)	< 0.001	5(18.5%)	1(0.9%)	0.001
Clavien-Dindo classification						
Grade I-II	1(3.1%)	30 (3.7%)		1(3.6%)	5(5.2%)	1.000
Grade III-IV	3 (9.4%)	27 (3.3%)		2(10.7%)	8(8.3%)	0.185
Complications			0.239			0.970
AL	3 (9.4%)	31 (3.8%)	0.116	1(3.7%)	1(0.9%)	0.368
Ileus	0 (0.0%)	8 (0.9%)	0.573	0(0.0%)	2(1.9%)	1.000
Wound infection	1 (4.0%)	6 (0.7%)	0.001	2(7.4%)	0(0.0%)	0.041
Intraperitoneal infection	0 (0.0%)	4 (0.5%)	0.691	0(0.0%)	1(1.0%)	0.588
Hemorrhage	0 (0.0%)	6 (0.7%)	0.626	0(0.0%)	3(2.8%)	1.000
Urinary infection	0 (0.0%)	2 (0.2%)	0.779	0(0.0%)	0(0.0%)	1.000
Mortality	0 (0.0%)	0 (0.0%)	1.000	0(0.0%)	0(0.0%)	1.000
Time-to-first exhaust	3(2,4)	3 (3, 4)	0.079	3(2,4)	3 (3, 4)	0.162
Time-to-first oral intake	4(3,4.75)	3 (3, 4)	0.925	4(3,5)	3 (3, 4)	0.387
Length of stay	9(7.25,11)	9 (8, 11)	0.552	9(8,12)	9(8,11)	0.384

*AR* Anterior resection, *LAR* Low anterior resection, *LCA* Left colic artery, *AL* Anastomotic leakage

^*^The Hartmann procedure was mandatorily performed due to intestinal malformation or marginal arch injury

^a^Forced to perform the Hartmann procedure and postoperative anastomotic leakage

After matching, three patients in the PDM group and one in the non-PDM group needed conversion to open surgery or extracorporeal IMA dissection (11.1% vs. 0.9%, *p* = 0.008). The median operative time for the PDM group was longer than that for the non-PDM group (210 vs. 163 min, *p* = 0.001). Furthermore, the median intraoperative blood loss in the PDM group was significantly more than that in the non-PDM group (50 vs. 30 mL, *p* = 0.002). The marginal arch injury (14.8% vs. 0.9%, *p* = 0.006), the ratio of free splenic flexure (22.2% vs. 3.8%, *p* = 0.005), and the rate of mandatory Hartmann procedure (18.5% vs. 0.0%, *p* < 0.001) were significantly higher in the PDM than those in the non-PDM group. The proportion of anastomotic failure in the PDM group was significantly higher than that in the non-PDM group (18.5% vs. 0.9%, *p* = 0.001). Moreover, anastomotic leakage (AL) incidence in the PDM group displayed an increasing tendency (3.7% vs. 0.9%, *p* = 0.368). Wound infection incidence in PDM group raised than that in non-PDM group (7.4% vs. 0.0%, *p* = 0.041).

### Risk factors for prolonged operative time and anastomotic failure

We further explored PDM’s impact on the prolonged operative time and anastomotic failure. Variables that may affect operative time and anastomotic failure were included. Univariate analysis revealed that Hartmann procedure (OR: 0.097, 95% CI:0.011–0.837, *p* = 0.034), and PDM (OR:4.084,95% CI:1.658–8.720, *p* = 0.001) were the factors affecting the operative time (Table 4). In addition, we observed PDM (OR: 3.205,95% CI:1.457–7.049, *p* = 0.004) as an independent risk factors affecting operative time using further multivariate analysis. The factors that may lead to anastomotic failure were also analyzed (Table 5). Univariate analysis revealed that age (OR:0.472, 95% CI: 0.235–0.948 *p* = 0.035), nCRT (OR:2.477, 95% CI:1.195–5.133, *p* = 0.017), LCA retention (OR:0.433, 95% CI:0.204–0.916, *p* = 0.029), and PDM (OR:9.81, 95% CI:3.817–25.213, *p* < 0.001) were the risk factors for anastomotic failure. Finally, multivariate analysis proved that nCRT (OR:2.782, 95% CI: 1.076–7.193, *p* = 0.038) and PDM (OR:7.601, 95% CI: 2.245–25.729, *p* = 0.003) were the independent risk factors for anastomotic failure.

**Table 4:** Univariate and multivariate regression analyses of operative time in 845 patients

Variables	Univariate analysis		Multivariate analysis	
	OR (95% CI)	*P-value*	OR (95% CI)	*P-value*
Age (≥ 60 / < 60 years)	0.996 (0.748–1.327)	0.978		
Gender (male/female)	1.196 (0.892–1.604)	0.231		
BMI (≥ 25/ < 25 kg/m^2^)	1.269 (0.947–1.699)	0.110		
ASA (I-II/III)	0.923 (0.607–1.404)	0.708		
nCRT (yes/no)	1.433 (0.972–2.112)	0.069		
Surgery procedure (Colorectal anastomosis /Hartmann)	0.098 (0.011–0.839)	0.034	0.263 (0.27–2.573)	0.251
LCA retention (yes/no)	1.463 (0.982–2.181)	0.062		
CEA (≥ 5/ < 5 ng/L)	1.005 (0.738–1.368)	0.976		
T stage (T0-T2/T3-T4)	0.955 (0.316–2.886)	0.770		
N stage (N0/N1-N2)	1.089 (0.777–1.527)	0.653		
M (0/1)	1.068 (0.538–2.121)	0.886		
PDM (yes/no)	4.084 (1.658–8.720)	0.001	3.205 (1.457–7.049)	0.004

*OR* Odds ratio, *CI* Confidence interval, *BMI* Body mass index, *ASA* American Society of Anesthesiologists, *CEA* Carcinoembryonic antigen, *nCRT* Neoadjuvant chemoradiotherapy, *LCA* Left colic artery, *PDM* Persistent descending mesocolon

**Table 5:** Univariate and multivariate regression analyses of anastomosis failure in 845 patients

Variables	Univariate analysis		Multivariate analysis	
	OR (95% CI)	*P-value*	OR (95% CI)	*P-value*
Age (≥ 60/ < 60 years)	0.472 (0.235–0.948)	0.035	0.505 (0.246–1.038)	0.063
Gender (male/female)	1.781 (0.853–3.717)	0.127		
BMI (≥ 25/ < 25 kg/m^2^)	1.293 (0.672–2.490)	0.442		
ASA (I-II/III)	0.331 (0.079–1.393)	0.132		
Tumor location (sigmoid/rectal)	0.710 (0.362–1.392)	0.319		
nCRT (yes/no)	2.477 (1.195–5.133)	0.017	2.782 (1.076–7.193)	0.038
LCA retention (yes/no)	0.433 (0.204–0.916)	0.029	0.547 (0.252–1.380)	0.187
CEA (≥ 5/ < 5 ng/L)	0.886 (0.433–1.814)	0.741		
T stage (T0-T2/T3-T4)	0.222 (0.013–3.744)	0.297		
N stage(N0/N1-N2)	1.087 (0.503–2.350)	0.831		
M (0/1)	1.873 (0.549–6.389)	0.316		
PDM (yes/no)	9.810 (3.817–25.213)	< 0.001	7.601 (2.245–25.729)	0.003

*OR* Odds ratio, *CI* Confidence interval, *BMI* Body mass index, *ASA* American Society of Anesthesiologists, *CEA* Carcinoembryonic antigen, *nCRT* Neoadjuvant chemoradiotherapy, *LCA* Left colic artery, *PDM* Persistent descending mesocolon

## Discussion

**Fig. 5:**
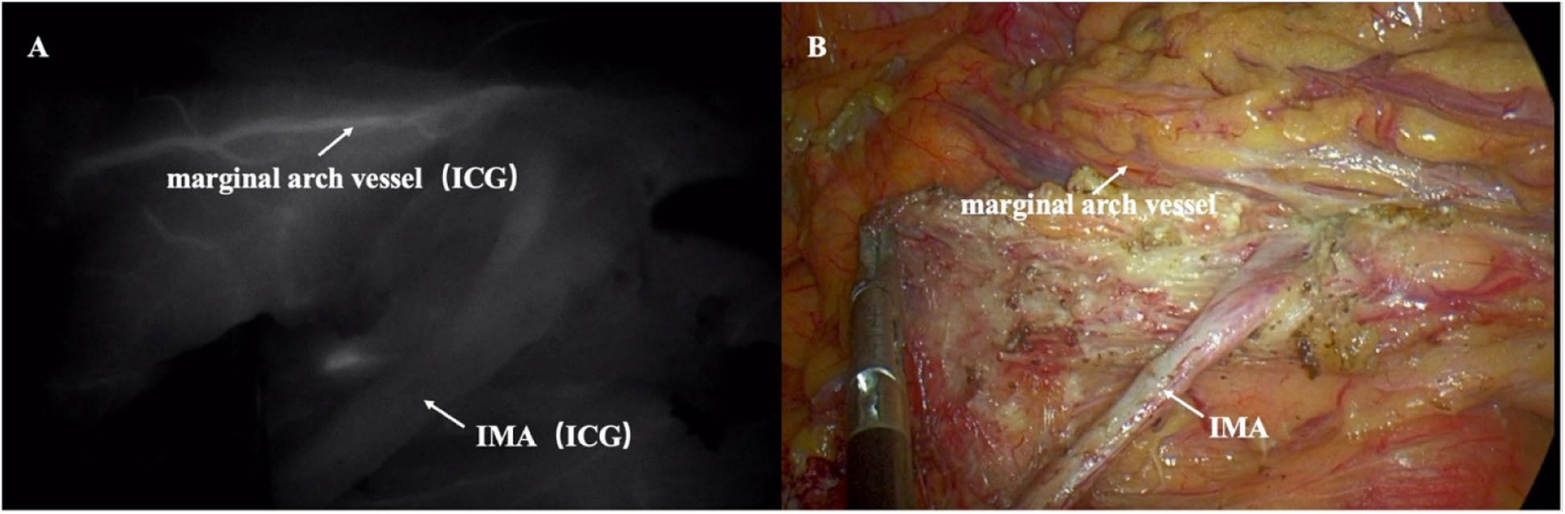
Intraoperative ICG fluorescent angiography presented IMA type IV (LCA lacking). **A** Monochrome NIR images; **B** White-light images

With the gradual unification and recognition of PDM diagnostic criteria, more colorectal surgeons have realized the influence of PDM on laparoscopic colorectal surgery [16]. The research on PDM mainly focuses on articles published by scholars in recent years. Wang et al. reviewed the records of 2775 patients who underwent laparoscopic radical colorectal cancer surgery at a single center [17]. Sixty patients (2.1%) were diagnosed with PDM: five were diagnosed preoperatively, and the others were diagnosed during surgery. Hanaoka et al. investigated PDM's frequency and radiological features for left-sided colorectal cancer with a ratio of 2.3% [18]. In our study, the frequency of PDM in the 845 cases was 3.8%. The lengths from IMA to IMV, IMA to the marginal arch, and IMA to the colon were significantly shorter in the PDM group (*p* < 0.001). PDM was an independent risk factor for prolonged operative time (OR = 3.205, *p* = 0.004) and anastomotic failure (OR = 7.601, *p* = 0.003). To the best of our knowledge, this study is the first to reveal that PDM was an independent risk factor for increased operative time and anastomotic failure for SRCs being subjected to radical surgery. However, the radiological diagnostic criterion has not completely reached a consensus. Porky et al. first described the imaging manifestations of PDM using barium and air-contrast enema examinations in 1966 [19]. Barium enema revealed that the sigmoid colon was longer and moved to the right side of the abdominal cavity, adjacent to the ileocecal mesentery, and the descending colon moved to the middle of the abdominal cavity. The CT axial scan revealed PDM when the right margin of the descending colon was located medially to the left renal hilum. Hanaoka et al. further revealed that the median lengths between the IMA and IMV and the descending colon in PDM cases were 14.8 mm and 17.2 mm, respectively, significantly shorter than those in non-PDM cases. In this study, more variables, including the length and shift of IMA; IMA type; and the lengths between the IMA and IMV, IMA and LCA, IMA and the marginal arch, and IMA and the descending colon were measured using MPR and MIP in 845 cases. Short lengths from IMA to IMV, IMA to LCA, and IMA to the colon [20]. Besides, the IMA to marginal arch length was significantly shorter in the PDM than that in the non-PDM group (2.8 vs. 8.4 cm, *p* < 0.001), which is important imaging evidence that the marginal artery is more prone to injury after IMA ligation. However, caution should be taken not to damage the marginal arch, and if necessary, intraoperative ICG fluorescence angiography can be used to determine vessel distributions [21] (Fig. 5A, B).

PDM was an independent risk factor for prolonged operative time (OR = 3.205, *p* = 0.004); nevertheless, some factors may increase the difficulty for PDM patients. First, the midline shift of the descending colon and extensive intraperitoneal adhesions are the main features of PDM. The sigmoid colon adhesions in most PDM patients are in the right lower abdomen but not the left. Some adhesion to the right pelvic wall and the ileocecal mesentery block IMA exposure [22]. Therefore, if the operator stands on the right side, it increases the difficulty and leads to more time being needed to separate the adhesions in the counter-direction views. Second, the mesentery of the sigmoid colon was not fully expanded, and the length from IMA to the colon was significantly shorter in the PDM group (3.5 vs. 9.8 cm, *p* < 0.001). After completing the separation of congenital adhesions, we proposed the sequence of dissociating the rectum prior to IMA ligation. The right yellow-white junction line cuts into the retrorectal space and is fully exposed to the opposite side. After joining the left and right sides, a sling was used to bind the rectum and mesentery, lift it from the caudal to the cranial direction to expand the retrorectal space, and finally reach the IMA root. Third, vascular IMA variations and their branches increase the difficulty of IMA, especially during LCA preservation. The lengths from IMA to IMV (1.6 vs. 2.5 cm, *p* < 0.001) and IMA to the marginal arch (2.8 vs. 8.4 cm, *p* < 0.001) were significantly shorter in the PDM group. The increased incidence of marginal vascular arch injuries and splenic flexure dissection will inevitably increase the surgery difficulty and prolong the operation time. Wang et al. revealed that the operative time of the PDM group was significantly longer (217.7 vs. 176.2 min, *p* = 0.003), and the volume of blood loss was higher (32.3 vs. 16.7 mL, *p* = 0.03). However, Hanaoka et al. did not observe similar results (176.0 vs. 171.5 min, *p* = 0.755).

Our multivariate analyses results revealed that PDM was an independent risk factor for anastomotic failure (HR = 7.601, *p* = 0.003). Furthermore, AL incidence in the PDM group displayed an increasing tendency (3.7% vs. 0.9%), though the difference was not significant. Due to the variation of the mesentery and IMA in PDM, the blood supply at the proximal end of the anastomosis is easily weakened. Mesenteric contracture can cause increased anastomotic tension, thereby affecting AL occurrence. In contrast, the descending colon is often medially located. Therefore, when the marginal artery was injured, the splenic flexure was freed, the proximal bowl extension was limited, and the Hartmann procedure had to be performed. Wang et al. revealed that rates of postoperative AL did not differ in both groups. LCA retention had no impact on proximal specimen margins or AL in patients with PDM. Hanaoka et al. also presented similar postoperative complications (8.0% vs. 10%, *p* = 0.346) but with a small sample size.

The limitations of this study lie in the retrospective study design and patient selection bias. Furthermore, some patients with congenital malformation of the descending colon that does not meet the diagnostic criteria for PDM might cause deviations in the significance of the results, and the cases of PDM observed during operation might cause deviations in the significance of the results. Moreover, the relatively small PDM sample and lack of long-term oncological outcomes affected the results. However, our study used the largest PDM series to date with detailed radiological data and operative imaging and was the first to evaluate CT imaging using MPR and MIP [23]. Meanwhile, more prospective and multicenter studies need to be done.

## Conclusion

In conclusion, PDM with mesenteric and IMA variation is an independent risk factor for prolonged operative time and anastomotic failure. Therefore, preoperative evaluation using MPR and MIP can help surgeons handle this rare congenital variant better.

## Data Availability

The datasets generated during and/or analyzed during the current study are not available.

## Abbreviations

PDM: Persistent descending mesocolon
SRCs: Sigmoid and rectal cancers
MRP: Multiplanar reconstruction
MIP: Maximum intensity projection
IMA: Inferior mesenteric artery
IMV: Inferior mesenteric vein
LCA: Left colic artery
AR: Anterior resection
LAR: Low anterior resection
nCRT: Neoadjuvant chemoradiotherapy
HR: Hazard ratio

## References

[1] Morgenstern L (1960). Persistent descending mesocolon. Surg Gynecol Obstetr.

[2] Nozawa H, Okamoto K, Kawai K, et al. Anatomical features of inferior mesenteric and left colic arteries and surgery in colorectal cancer patients with persistent descending mesocolon. ANZ J Surg. 2022;92:1760–65. 10.1111/ans.17683 35412011

[3] Ghahremani GG (2022). Radiological features and clinical implications of persistent congenital mesocolon: Pictorial essay. J Med Imaging Radiation Oncol..

[4] Hamada K, Sumida Y, Ozeki K (2022). Persistent descending mesocolon as an intraoperative risk factor in laparoscopic surgery for left-sided colon and rectal cancer. Asian J Endosc Surg.

[5] Mochizuki T, Tazawa H, Hirata Y (2017). A colovesical fistula with a persistent descending mesocolon due to partial situs inversus: A case report. Int J Surg Case Rep.

[6] Kawakami M, Nakazato H, Tomiyama T (2020). Laparoscopic sigmoidectomy for sigmoid colon cancer with left-sided inferior vena cava and persistent descending mesocolon. J Surg Case Rep.

[7] Chen A, Yang FS, Shih SL (2003). Case report. CT diagnosis of volvulus of the descending colon with persistent mesocolon. AJR Am J Roentgenol..

[8] Chang YT, Lee JY, Liao YM (2008). Laparoscopic resection of a giant retroperitoneal T-shaped duplication of descending colon. J Pediatr Surg.

[9] Hisano K, Ueki T, Kono H (2019). Laparoscopic high anterior resection for triple colorectal cancers with persistent ascending and descending mesocolons: A case report. Asian J Endosc Surg.

[10] Mitchell A, Dugas A (2019). Malakoplakia of the colon following renal transplantation in a 73 -year-old woman: report of a case presenting as intestinal perforation. Diagn Pathol.

[11] Matsuo T, Otsuka K, Kimura T (2021). Laparoscopic colectomy for persistent descending mesocolon in sigmoid colon cancer: a case report. Int J Surg Case Rep.

[12] Bueno MR, Estrela C, Granjeiro JM (2021). Cone-beam computed tomography cinematic rendering: clinical, teaching and research applications. Braz Oral Res.

[13] Murono K, Kawai K, Kazama S (2015). Anatomy of the inferior mesenteric artery evaluated using 3-dimensional CT angiography. Dis Colon Rectum.

[14] Zheng MH, Ma JJ, Zang L (2015). Laparoscopic middle cephalic approach for radical resection of rectal cancer. Chin J Gastrointest Surg (in Chinese).

[15] Clavien PA, Barkun J, de Oliveira ML (2009). The Clavien-Dindo classification of surgical complications: five-year experience. Ann Surg.

[16] Shetty P, Nayak SB (2014). Absence of transverse colon, persistent descending mesocolon, displaced small and large bowels: a rare congenital anomaly with a high risk of volvulus formation. Anatomy Cell Biol.

[17] Wang L, Kondo H, Hirano Y (2020). Persistent descending mesocolon as a key risk factor in laparoscopic colorectal cancer surgery. In Vivo.

[18] Hanaoka M, Hino H, Shiomi A (2021). Minimally invasive surgery for colorectal cancer with persistent descending mesocolon: radiological findings and short-term outcomes. Surg Endosc.

[19] Popky GL, Lapayowker MS (1966). Persistent descending mesocolon. Radiology.

[20] Serena G, Nardi L, Schmeisser MJ (2021). Carl Toldt centennial, surgeon and anatomist. Am Surg.

[21] Furuichi Y, Kumamoto K, Asano E (2020). Four cases of laparoscopic colectomy for sigmoid colon and rectal cancer with persistent descending mesocolon. Surg Case Rep.

[22] Blanco-Colino R, Espin-Basany E (2018). Intraoperative use of ICG fluorescence imaging to reduce the risk of anastomotic leakage in colorectal surgery: a systematic review and meta-analysis. Tech Coloproctol.

[23] Hisano K, Ueki T, Kono H (2018). Laparoscopic high anterior resection for triple colorectal cancers with persistent ascending and descending mesocolon: a case report. Asian J Endosc Surg.

